# Risk Factors Associated with Ebola and Marburg Viruses Seroprevalence in Blood Donors in the Republic of Congo

**DOI:** 10.1371/journal.pntd.0003833

**Published:** 2015-06-05

**Authors:** Nanikaly Moyen, Laurence Thirion, Petra Emmerich, Amelia Dzia-Lepfoundzou, Hervé Richet, Yannik Boehmann, Yannick Dimi, Pierre Gallian, Ernest A. Gould, Stephan Günther, Xavier de Lamballerie

**Affiliations:** 1 Aix Marseille University, IRD French Institute of Research for Development, EHESP French School of Public Health, EPV UMR_D 190 "Emergence des Pathologies Virales", Marseille, France; 2 Centre National de Transfusion Sanguine, Brazzaville, Republic of Congo; 3 IHU Méditerranée Infection, APHM Public Hospitals of Marseille, Marseille, France; 4 Department of Virology, Bernhard-Nocht-Institute for Tropical Medicine, Hamburg, Germany; 5 Etablissement Français du Sang Alpes Méditerranée, Marseille, France; U.S. Naval Medical Research Unit Six, UNITED STATES

## Abstract

**Background:**

Ebola and Marburg viruses (family *Filoviridae*, genera *Ebolavirus* and *Marburgvirus*) cause haemorrhagic fevers in humans, often associated with high mortality rates. The presence of antibodies to Ebola virus (EBOV) and Marburg virus (MARV) has been reported in some African countries in individuals without a history of haemorrhagic fever. In this study, we present a MARV and EBOV seroprevalence study conducted amongst blood donors in the Republic of Congo and the analysis of risk factors for contact with EBOV.

**Methodology and Findings:**

In 2011, we conducted a MARV and EBOV seroprevalence study amongst 809 blood donors recruited in rural (75; 9.3%) and urban (734; 90.7%) areas of the Republic of Congo. Serum titres of IgG antibodies to MARV and EBOV were assessed by indirect double-immunofluorescence microscopy. MARV seroprevalence was 0.5% (4 in 809) without any identified risk factors. Prevalence of IgG to EBOV was 2.5%, peaking at 4% in rural areas and in Pointe Noire. Independent risk factors identified by multivariate analysis were contact with bats and exposure to birds.

**Conclusions/Significance:**

This MARV and EBOV serological survey performed in the Republic of Congo identifies a probable role for environmental determinants of exposure to EBOV. It highlights the requirement for extending our understanding of the ecological and epidemiological risk of bats (previously identified as a potential ecological reservoir) and birds as vectors of EBOV to humans, and characterising the protection potentially afforded by EBOV-specific antibodies as detected in blood donors.

## Author Summary

Ebola and Marburg viruses cause haemorrhagic fevers often fatal to humans. Here, we looked for antibodies to Ebola and Marburg viruses (*i*.*e*., markers of previous contact with these viruses) in Congolese blood donors with no previous history of haemorrhagic fever. We found serologic evidence for contact with Marburg and Ebola viruses in 0.5% and 2.5% of blood donors, respectively. The circulation of Marburg virus occurs at a very low rate without any identified risk factor. In contrast, prevalence to Ebola virus was peaking at 4% in rural areas and in Pointe Noire city. Importantly, we identified that contacts with bats and birds constituted two independent environmental determinants of exposure. This study confirms that contact with Ebola virus is not infrequent in Congo and can occur in the absence of haemorrhagic fever. It highlights the requirement for further investigating the role of bats and birds in the ecological cycle of Ebola, and for determining whether asymptomatic contact with Ebola virus can provide subsequent protection against severe forms of the Ebola disease.

## Introduction

Marburg and Ebola viruses (family *Filoviridae*, genera *Marburgvirus* and *Ebolavirus*) cause severe Viral Haemorrhagic Fever (VHF) in humans, with a high fatality rate in symptomatic cases [1,2,3]. They appear to infect and persist in some species of fruit bats, that may serve as natural reservoirs for these viruses [4,5,6,7,8,9]. Non-human primates have been a source of human infections however they are not thought to be the reservoir as they develop severe, fatal illness when infected [10].

The genus *Marburgvirus* currently consists of a single species *Marburg marburgvirus*, of which the recognised members are *Marburg virus* (MARV) *and Ravn virus* [11,12]. The first cases of Marburg haemorrhagic fever (MHF) occurred in Germany and Serbia (in the former Yugoslavia) in 1967 and were linked to laboratory work using tissues dissected from African green monkeys imported from Uganda [13,14]. The first major outbreak of MHF occurred in Democratic Republic of the Congo (DRC, formerly Zaire), from 1998 to 2000 [15,16]. A second, even more devastating outbreak occurred in Angola in 2004–2005 with a reported case fatality rate (CFR) of almost 90% [17,18].

In Nigeria and DRC, seroprevalence studies identified antibodies to MARV in less than 2% of apparently healthy people selected in general population in Nigeria and amongst healthcare workers and general population in DRC [19,20]. In the Central African Republic (CAR), antibodies to MARV were observed in both Pygmy (0.7–5.6%) and non-Pygmy (0.0–3.9%) populations [21]. An African serosurvey of VHF (Crimean-Congo haemorrhagic fever, Rift Valley fever, Lassa, Hantaan, EBOV and MARV), conducted in the 1980s in the Central African general population, reported low prevalence values: 0.3% in N’Djamena (Tchad), 2.6% in Bioco Island (Equatorial Guinea) and, in the Republic of Congo, 3% in Pointe-Noire but no seropositive sera to MARV detected in people in Brazzaville [22]. To date, no case of MHF has been reported in the Republic of Congo.

The genus *Ebolavirus* includes five species: *Zaire ebolavirus* (Ebola virus: EBOV), *Sudan ebolavirus*, *Taï Forest ebolavirus*, *Reston ebolavirus* and *Bundibugyo ebolavirus* [11,12]. The genus *Ebolavirus* is primarily African in origin, with the exception of the species *Reston ebolavirus* which is Asian [23]. EBOV was first identified in 1976, in Southern Sudan [24] and in the North of DRC [25,26]. Since then, outbreaks have been described in several other African countries (the Republic of Congo, Ivory Coast, DRC, Gabon, Sudan, Uganda, Guinea, Sierra Leone and Liberia) [1,27,28,29,30,31,32,33,34], with reported (CFR) frequently exceeding 50% amongst symptomatic patients. In the Republic of Congo where the current study took place, several outbreaks of (Zaire) EBOV were reported in the North of the country (2001 in Olloba-Mbomo, 2002 in Kéllé, 2003 in Mbandza-Mbomo), with 75 to 89% reported fatality rates [35,36,37].

In previous seroprevalence studies, amongst 1,517 apparently healthy persons tested in five regions of the Cameroon, a positive rate of 9.7% was found with highest rates amongst Pygmies (14.5%), young adults (11.6%) and rain forest farmers (13%) [38]. In CAR, the seropositivity rate was 5.3% and Pygmies appeared to have a higher seroprevalence than non-Pygmies (7% *versus* 4.2%) [21]. During the 1995 outbreak of Ebola virus disease in the region of Kikwit (Democratic Republic of Congo), villagers had a greater chance of exposure (9.3%) than forest and city workers (2.2%) [39]. In a large study conducted in 220 villages in Gabon (4,349 individuals enrolled), antibodies against EBOV were detected in 15.3% of those tested, with the highest levels in forested regions (17.6% and 19.4% respectively in forest and deep forest areas), suggesting the occurrence of mild or asymptomatic infections [40,41]. In the Republic of Congo, seroprevalence values reported in the late 1980's were 7.8% in Pointe-Noire and 6.2% in Brazzaville [22].

In Sierra Leone, in 2006–2008, among 253 febrile patients negative for Lassa fever and malaria, antibodies against EBOV and MARV were detected in respectively 8.2% et 3.2% of the samples [42].

In this study, we present an analysis of MARV and EBOV seroprevalence amongst blood donors in the Republic of Congo in 2011 and we report associated risk factors for contact with EBOV.

## Materials and Methods

### Study Design

A MARV and EBOV seroprevalence study was performed in 2011 in the Republic of Congo, using a prospective cohort of blood donors.

### Setting

**Fig 1:**
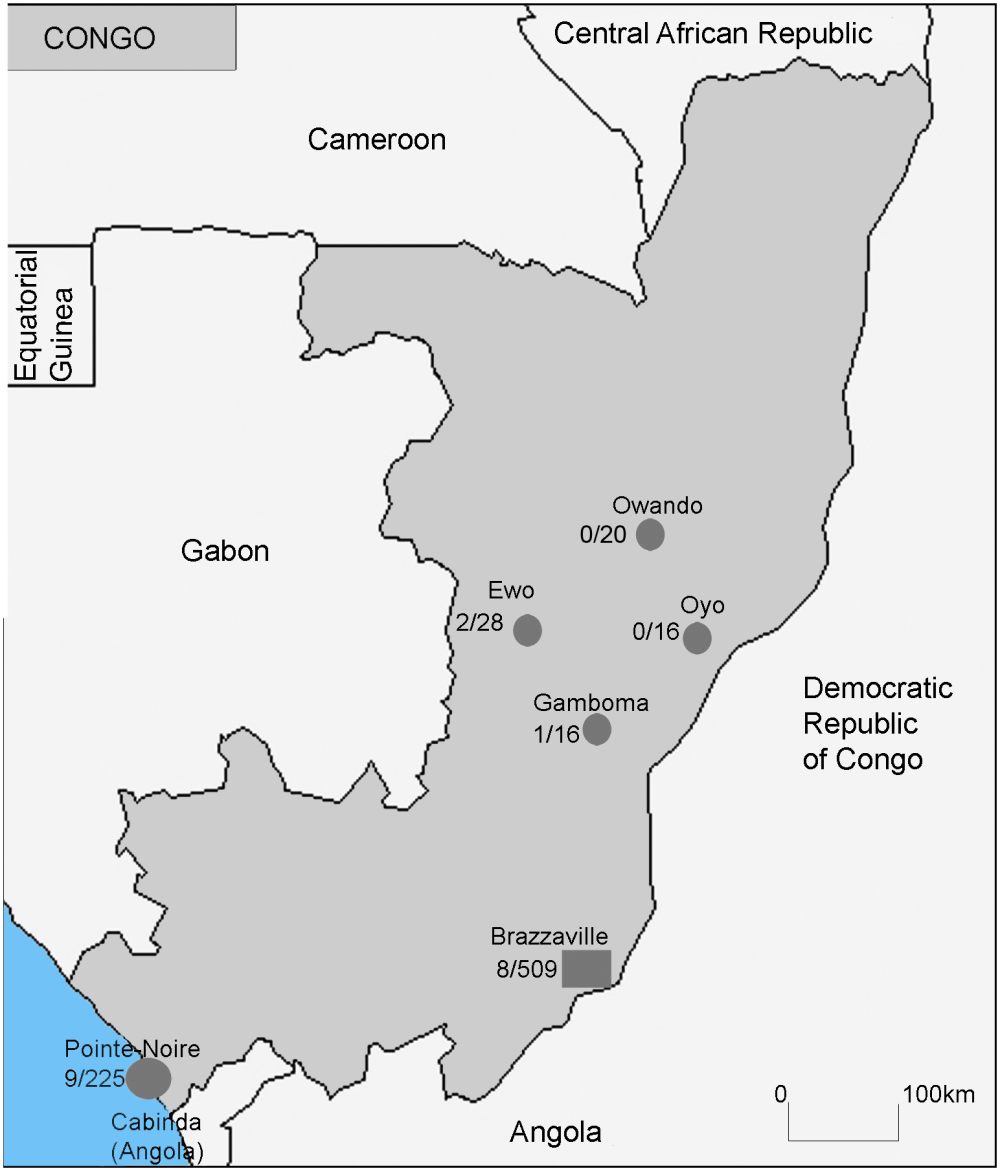
Map of Congolese study sites. Rural locations and Pointe-Noire city (circles); Brazzaville, the capital (square). Brackets: number of positive samples/ total of samples.

Field samples for the study were collected from March to July 2011, in the Republic of Congo (Fig 1) in urban areas (Brazzaville and Pointe-Noire) and in rural locations (Gamboma, Owando, Oyo and Ewo). Ewo is the capital of the Department of Cuvette-Ouest, where all previous EBOV outbreaks occurred.

This study was performed in collaboration with the Centre National de Transfusion Sanguine (CNTS) of Congo; the Virology Laboratory UMR_D 190 "Emergence des Pathologies Virales" (Aix-Marseille University, IRD French Institute of Research for Development, EHESP French School of Public Health), Marseille, France and the Virology Laboratory of Bernhard-Nocht-Institut für Tropenmedizin, Hamburg, Germany.

### Population Studied

Blood donors of both genders were included. The criteria for enrollment were eligibility for blood donation and provision of informed consent without specific limiting factors. The age of blood donors ranged from 18 to 65 years.

### Ethical Considerations

Serum samples for serological analyses were collected in collaboration with the CNTS. Informed, written consent was obtained from each person enrolled in the study and the consent procedure was approved by the Congolese Research in Health Sciences Ethics Committee (N° 00000065 DGRST/CERSSA).

### Data Collection

A structured questionnaire was administered face-to-face, in the official language (French) and/or in national languages (Lingala or Kutumba). All questionnaires were completed by the medical personnel conducting the interviews.

The following data were collected: socio-demographic circumstances, domestic characteristics (age, gender, occupation, residence, size of household, type of house, water resource, usage of mosquito nets), environmental characteristics (animal contacts and/or consumption), travel outside the country during their lifetime, history of haemorrhagic fever (in family or personal).

### Serum

Venous blood samples were drawn using two 4 mL plain tubes which were immediately centrifuged. Sera were kept at -80°C until use. Aliquots were inactivated at 56°C for 30 min and transferred to the Virology Laboratory of the Bernhard-Nocht-Institut (BNI) in Hamburg for serological assays.

### Serological Tests

Serum IgG antibodies specific for EBOV and MARV were titrated using indirect double-immunofluorescence microscopy assays which were recorded as positive if reciprocal end point titres were ≥20 [43].

Antigens consisted of acetone-fixed Vero cells infected with Ebola virus (strain ATCC 1978) or Marburg virus (strain Popp 1967). Cultivation of the viruses was carried out in an approved and compliant BLS4 laboratory in BNI.

Serum samples were tested as serial twofold dilutions from 1:20 to ≥ 1:160 and antibodies were detected with a Fluorescein isothiocyanate (FITC) labelled anti-human IgG antibody-conjugate. Cell smears were counterstained with specific anti-Ebola or anti-Marburg nucleocapsid monoclonal antibodies (provided by the Institute of Virology, University of Marburg) using a rhodamine-anti-mouse conjugate as secondary antibody [44]. As positive controls for Ebola virus, we used *(i)* a human polyclonal antibody for the first IF with a titre at 2,560 (IgG) and *(ii)* a mouse monoclonal antibody with a titre at 1,280 (IgG). As positive controls for Marburg virus, we used *(i)* a human polyclonal antibody for the first IF with a titre at 1,280 (IgG) and (ii) a mouse monoclonal antibody with a titre at 640 (IgG).

This “double immunofluorescence” protocol provides a much higher specificity than regular immunofluorescence assays, since only antibodies that detect filoviral antigens in co-localisation with a monoclonal antibody are considered.

### Statistical Analysis

Statistical analyses were performed using the IBM SPSS statistic 21 software. Analyses aiming at analysing risk factors for seropositivity included univariate, stratified and multivariate analyses. The Fisher’s exact test was used to compare proportions in univariate analysis and the ANOVA test to compare means. The Pearson’s test was used for stratified analysis.

All statistical analyses were performed at the 95% confidence level. The association between anti-EBOV IgG seropositivity and risk factors was determined by binary logistic regression analysis. Stratified analysis based on sex, age and area were performed. The significant variables in univariate analysis were entered in the multivariate model. The quality of the multivariate model was assessed with Hosmer-Lemeshow’s test.

## Results

Sociodemographic characteristics are presented in Table 1. Overall, 809 blood donors provided serum samples; 734 (90.7%) lived in urban areas (62.9% and 27.8% in Brazzaville and Pointe-Noire, respectively), 370 (45.7%) of which were younger than 30 years old (yo) with a median age of 31. The gender ratio (Male/Female) was 3.02. Most blood donors lived in a modern house 723 (89.4%). The two most common occupations were “student” (20.9%) and “unemployed” (26.5%).

**Table 1:** Socio-demographic characteristics of blood donors in study areas, March-July 2011.

	Study population N = 809	General population N = 24,509
**Residence; No. (%)**		
Brazzaville	509 (62.9)	11,962 (48.8)
Pointe-Noire	225 (27.8)	10,014 (40.9)
Rural areas	75 (9.3)	2,533 (10.3)
**Gender**		
Male/Female	608/201	18,906/5,603
Gender ratio	3.02	3.37
**Age, y**		
Mean	32.89	-
Median	31	-
Range	18–63	18–65
SD	10.21	-
**Age groups; No. (%)**		
≤ 29 y	370 (45.7)	-
30–39 y	238 (29.4)	-
40–49 y	130 (16.1)	-
50+ y	71(8.8)	-
**Occupation; No. (%)**		
Health professional	88 (10.9)	-
Office worker	64 (7.9)	-
Military	105 (13.0)	-
Unemployed	214 (26.5)	-
Student	169 (20.9)	-
Hunter	17 (2.1)	-
Cultivator	15 (1.9)	-
Farmer	17 (2.1)	-
Manual labourer	120 (14.8)	-
**Household size; No. (%)**		
1–5	439 (54.3)	-
6–10	303 (37.5)	-
≥ 11	67 (8.3)	-
**Type of house; No. (%)**		
Modern	723 (89.4)	-
Traditional	86 (10.6)	-

Epidemiological analyses of EBOV and MARV infections were performed separately. However, the same potential risk factors for seropositivity (gender, age, household size, occupation, mosquito net, travel risk, exposure to rodents/forests animals, and consumption of forests animals) were assessed for both viruses.

Individual titres of the MARV and EBOV-positives are presented in Table 2.

**Table 2:** Individual titres of the EBOV and MARV-positives.

Number of positive samples	Sites	IgG Titre
**MARV**		
1	Brazzaville	80
2	Brazzaville	80
3	Brazzaville	160
4	Brazzaville	160
**EBOV**		
1	Brazzaville	>160
2	Brazzaville	80
3	Brazzaville	40
4	Brazzaville	>160
5	Brazzaville	>160
6	Brazzaville	160
7	Brazzaville	80
8	Brazzaville	80
9	Pointe-Noire	80
10	Pointe-Noire	80
11	Pointe-Noire	80
12	Pointe-Noire	>160
13	Pointe-Noire	40
14	Pointe-Noire	20
15	Pointe-Noire	160
16	Pointe-Noire	40
17	Pointe-Noire	40
18	Gamboma	80
19	Ewo	40
20	Ewo	40

### MARV-Specific IgG Seroprevalence

IgG to MARV was identified in 0.5% of the donors tested (4 in 809). Seropositivity could not be significantly associated with any of the risk factors investigated in the individual questionnaire. Antibodies to MARV were detected exclusively in male blood donors from Brazzaville who had all been in contact (touching and catching) with rodents (mice and rats), but this is a common feature in out cohort (>80% of the donors reported such contact). Three out of four were students (median age 23) and one (55yo) was an office worker. None had been in contact with (or had eaten) forest animals or bats. The titre of IgG in positive donor ranged from 80 to 160.

### EBOV-Specific IgG Seroprevalence

The overall prevalence of positivity for IgG when tested against EBOV was 2.5% (20 in 809). It was 1.6% (8 in 509) in Brazzaville, 4% (3 in 75) in the rural locations (Gamboma, Owando, Oyo and Ewo) and 4% (9 in 225) in Pointe-Noire.

### Univariate Analysis

Table 3 summarises the association between potential risk factors and anti-EBOV IgG seropositivity using univariate analysis. Amongst the populations studied there was no statistically significant relationship between gender (p = 0.79), age (p = 0.96), travel (p = 0.62), household size (p = 0.39), exposure to rodents (p = 0.63) and the presence of IgG to EBOV. However, being a hunter (p = 0.01) was a risk factor, whereas the other occupations showed no statistical significance in univariate analysis. The use of simple mosquito nets had a protective effect. Importantly, significant association with bat contact or eating birds (p values respectively of <0.001 and 0.01) was identified.

**Table 3:** Association between potential risk factors and anti-EBOV IgG seropositivity.

	Frequency; (%)		Univariate analysis		Multivariate analysis	
Variables	IgG +	IgG -	OR (95% CI)	p-value	OR (95% CI)	p-value
**Gender**				0.41		0.63
Male	16 (2.6)	592 (97.4)				
Female	4 (2.0)	197 (98.0)				
**Age groups**				0.96		0.36
18–29	10 (2.7)	360 (97.3)				
30–39	5 (2.1)	233 (97.9)				
40–49	3 (2.3)	127 (97.7)				
50+	2 (2.8)	69 (97.2)				
**Household size**				0.39		
1–5	12 (2.7)	427 (97.3)				
6–10	8 (2.6)	295 (97.4)				
≥ 11	0	67 (100.0)				
**Occupation**						
Health professional	0	88 (100)		0.09		
Office worker	2 (3.1)	62 (96.9)		0.47		
Military	3 (2.8)	103 (97.2)		0.49		
Unemployed	3 (1.4)	210 (98.6)		0.18		
Student	6 (3.6)	163 (96.4)		0.22		
Hunter	2 (11.8)	15 (88.2)	5.1 (1.3–20.6)	0.01		0.7
Cultivator	1 (6.7)	14 (93.3)		0.31		
Farmer	0	17 (100)		0.65		
Manual labourer	3 (2.5)	117 (97.5)		0.58		
**Type of house**				0.64		
Modern	18 (2.5)	705 (97.5)				
Traditional	2 (2.3)	84 (97.7)				
**Mosquito net (usage)**						
Simple	3 (0.9)	338 (99.1)	0.24 (0.1–0.8)	0.01		0.06
Impregnated	9 (4.5)	190 (95.5)		0.06		
**Travels**						
Central Africa	7 (3.1)	219 (96.9)		0.31		
West Africa	0 (0.0)	31 (100.0)		0.45		
Other countries	0 (0.0)	31 (100.0)		0.45		
**Exposure to Rodents**				0.58		
Mice	17 (2.5)	669 (97.5)		0.638		
Rats	9 (3.3)	260 (96.7)		0.18		
**Exposure to Forest animals**						
Bats	7 (14.0)	43 (86.0)	8.17 (3.4–19.6)	<0.001	7.5 (2.6–21.7)	<0.001
Monkeys	2 (8.0)	23 (92.0)		0.12		
Birds	2 (4.1)	47 (95.9)		0.34		
Antelopes	2 (8.3)	22 (91.7)		0.11		
Snakes	1 (3.7)	26 (96.3)		0.49		
**Consumption of Forest Animals**						
Bats	0 (0.0)	23 (100.0)		0.55		
Monkeys	12 (3.1)	380 (96.9)		0.20		
Birds	10 (5.2)	182 (94.8)	3.21 (1.3–7.6)	0.01	2.6 (1.1–6.9)	0.04
Antelopes	12 (2.7)	436 (97.3)		0.42		
Snakes	3 (2.0)	146 (98.0)		0.48		

### Stratified Analysis

Seropositivity in age-groups was as follows: 2.7% in 18–29yo, 2.1% in 30–39yo, 2.3% in 40–49yo, 3.2% in 50–59yo and 0.0% in >60yo, with no evidence for an increase of seroprevalence with age. In the 18–29yo age-group, higher seroprevalence was associated with touching bats (p<0.001). In the second group (30–39yo), higher seroprevalence was associated with the military profession (p = 0.04). No significant association was found in the other age-groups. No significant association was identified in a stratified analysis by occupation, including military profession.

Regarding stratification by areas: in Pointe-Noire, to be a student (p = 0.002) or to have exposure to bats (p<0.001) was statistically associated with anti-EBOV IgG. Exposure to bats (p<0.001) was also found to be a risk factor in Brazzaville. Other variables (sex, age, household size, type of house, travel risk, exposure to rodents, and consumption of forest animals) were not significantly associated with the presence of IgG, regardless of the area in which donors lived.

Concerning stratification by gender (S1 Table), seropositivity was 2.6% in males and 2.0% in females. No significant association was identified in females. Amongst the male subpopulation, being a hunter (p = 0.009), having contact with bats (p<0.001) or monkeys (p = 0.01), or consuming birds (p = 0.002) was statistically associated with EBOV IgG positivity. Other variables (age, household size, type of house, travel risk, exposure to rodents) had no statistically significant relationship with the presence of IgG to EBOV.

### Multivariate Analysis

In the multivariate model (Table 3), the only variables independently associated with Ebola antibody detection were contact with bats (p<0.001) and bird consumption (p = 0.04).

## Discussion

Whilst the Democratic Republic of the Congo and neighbouring Angola have experienced Marburg disease outbreaks [2,45], no Marburg cases have been reported to date in the Republic of Congo. By contrast, human epidemics due to Ebola virus have been reported in the Republic of Congo in 2001, 2002 and 2003. All outbreaks were located in the Cuvette-Ouest Department [1,29,46].

Against this background, we conducted a seroprevalence study of Ebola and Marburg viruses amongst blood donors in the Republic of Congo, to estimate the seroprevalence of both viruses outside the epidemic period and also to identify possible risk factors. This study has some obvious limitations related to the population studied (*e*.*g*., the number of blood donors originating from rural areas was small, all participants were older than 18 years old and 75% were males) and to the capacity to collect epidemiological information without interfering with the process of blood donation (for example, travel inside the country and activities outside the main occupation were not documented). However a body of standardised information was obtained from the questionnaires.

Importantly, our biological analyses identified antibodies specific for Ebola virus (and to a lesser extent to Marburg virus) in healthy blood donors who did not report a history of haemorrhagic fever. This confirms previous studies suggesting that filoviruses can circulate in Africa in the absence of severe clinical presentations (*i*.*e*., associated with asymptomatic or mild infections) [47] and that Ebola virus-specific seroconversion can be observed without clinical manifestation [21] or possibly associated with atypical clinical presentation. In the case of Ebola virus infections, asymptomatic seropositives have been identified by different techniques (*e*.*g*., immunofluorescence [22] or ELISA [21]) and have been found more frequently in areas where Ebola cases were reported [40]. In addition, Leroy and collaborators detected the Ebola virus genome in white blood cells of asymptomatic seroconverters investigated for up to 3 weeks following exposure to documented Ebola symptomatic patients [41]. Contact with limited amounts of virus, specific infection routes [48], specific characteristics of individual immunity leading to production of specific IgG and early and strong inflammatory responses [47], may explain the presence or absence of symptomatic presentation. Finally, a recent study identified asymptomatic seropositives with antibodies against various linear epitopes located in different and both structural and non-structural EBOV proteins [49]. Altogether, this rules out the hypothesis that antibodies to EBOV detected in asymptomatic individuals in previous studies are massively false positives.

It has been proposed that some populations may have been exposed to yet unidentified virus strains that have relatively low pathogenicity for humans. Monath [50] proposed that pathogenic strains may have independent transmission cycles involving species rarely in contact with humans. In support of this hypothesis, Gonzalez [22] reported atypical serological responses in asymptomatic seropositives, consisting in identical titres against Ebola Mayinga from Zaire and Ebola Boniface from Sudan identified in 89% of cases. This hypothesis still stands but is weakened by *(i)* the fact that fifteen years of investigations of African wildlife species have failed to identify such low pathogenicity strains in host species in close contact with humans and *(ii)* the identification of asymptomatic seroconversions following contact with symptomatic patients [41].

Monath [50] further proposed that pathogenic strains may emerge from non-pathogenic strains by mutational events, but this hypothesis is weakened by the argument that the same strain of EBOV has been shown to be responsible for symptomatic cases and secondary asymptomatic cases [41].

In the late 1980's, Gonzalez and collaborators identified low MARV seroprevalence values in Pointe Noire and Brazzaville (3 and 0%, respectively) [22]. In our study, the prevalence of IgG to MARV was also low (0.5%) and could not be associated with specific risk factors. In contrast, the prevalence of antibodies to EBOV was 2.5%, peaking at 4% in rural populations and in the city of Pointe Noire. It was 1.6% in Brazzaville. Gonzalez and collaborators had previously reported 7.8 and 6.2% Ebola seroprevalence values in Pointe Noire and Brazzaville, respectively [22]. The differences observed may be explained by different technical protocols used for detection of antibodies, by the fact that studies were performed more than twenty years apart, and by the investigation of different populations since Gonzalez studied randomly selected clusters (providing representativeness of gender and age distribution). Against the latter hypothesis, it should be noted that, in both studies, no significant difference associated with age-group and gender could be identified.

Nevertheless, two remarkable aspects of our epidemiological observations deserve further discussion. On the one hand, antibodies to Ebola virus were detected in urban populations of Congolese blood donors, which should stimulate future investigations relating to asymptomatic seropositives. On the other hand, new environmental factors associated with seropositivity have been identified. In neighbouring Gabon, the seroprevalence was significantly higher in the forest regions (17.6%) than in savannah regions (10.5%) and Lakeland areas (2.7%) [40,48], but no sociodemographic or behavioural risk factors associated with EBOV seropositivity could be identified. Therefore, the finding in the current study that *(i)* exposure to bats and *(ii)* bird consumption are associated independently in multivariate analysis with an increased prevalence of antibodies to EBOV is of particular relevance.

The notion of bird consumption must be interpreted with caution. If birds are consumed, they will have been cooked, but bird consumption also implies contact with birds, either in the markets in Africa or when capturing them or preparing them for cooking. Whilst further studies to clarify the role of birds as a possible source of human infection are clearly justified, we prefer to refer to “exposure to birds” as a potential source of the detected seropositivity.

The possibility of exposure to birds, with supporting evidence, has never been described before as a risk factor for filovirus infection. To the best of our knowledge, birds have not been associated with EBOV or virus antigen in previous investigations [51]. In 1979 and 1980, a total of 1,664 animals (117 species, including 67 birds) were collected in the DRC and Cameroon during the dry season near the site of the 1976 Ebola haemorrhagic fever epidemic. This study failed to identify an animal reservoir of EBOV [52]. An ecologic investigation was also performed to identify the reservoir of EBOV following the 1995 outbreak in Kikwit, DRC. Most of the 3,066 collected specimens were mammals (87%, 2663), but birds (9%, 265) were also collected. All attempts at isolation of EBOV remained negative [53]. During a study in CAR (Ngotto forest) in 1998–99, 662 animals were trapped including 16 birds. Only *Zaire ebolavirus* nucleotide sequences were detected in six organs rodents [54]. Finally, experimental inoculation of birds with EBOV did not evidence significant acute or chronic replication [55].

In 1994, an outbreak of Ebola was described in a wild chimpanzee community in the Taï National Park, Ivory Coast. Before the beginning of the outbreak, chimpanzees spent a long time in a fig tree *(Ficus goliath)* that was full of fruits. Many birds (pigeons) were seen feeding on the figs during the day, and rodents and fruit bats were feeding on the tree during the night. It was proposed that the *F*. *goliath* could have constituted an epidemiological "hub", putting different species-including birds- in contact with one another [56].

The implication of birds in the epidemiology of filoviral disease was evoked by Monath [50] in a hypothetical transmission cycle, taking into consideration the possibility that filoviruses may be primary arthropod or plant viruses, transmitted to vertebrates including bats or other insectivorous species such as birds. Jeffers *et al*. [57] reported in 2002 a noticeable biochemical similarity between the glycoprotein of certain birds, oncogenic retroviruses and the EBOV glycoprotein. They suggested a possible common evolutionary origin which could be an indication that avian species have or have had a role in the ecology and evolution of filoviruses. Accordingly, the possible role of birds in the transmission of EBOV should not be ruled out and our intriguing observation suggests that additional studies to elucidate the ecology of filoviruses in birds would be of great interest.

The statistically significant identification of exposure to bats as an independent risk factor for EBOV infection is also remarkable since contact with bats has been reported as a risk factor for MARV infection [58,59,60,61], and fruit bats [6] (and possibly insectivorous bats [62]) are thought to have a significant role in the ecological cycle of EBOV. Therefore, the current study reinforces the suspected importance of bats in the natural cycle of Ebola virus and its possible transmission to humans.

### In Conclusion

Our results imply that in the Republic of Congo, the circulation of Marburg virus occurs at a very low rate without any identified risk factor, but that human exposure to Ebola virus without consequent disease is not infrequent. Living in Pointe Noire or in a rural area, and having contact with bats and birds is associated with a higher risk of exposure to Ebola virus. Unfortunately, little is known about the natural history and biological properties of EBOV antibody in individuals without haemorrhagic fever. Here, we did not observe an increase of seroprevalence with age, which may suggest that, in some individuals, the antibody titre decreases and becomes undetectable with time. This would in turn imply the absence of iterative contacts with Ebola virus antigens or that of a strong antibody response following such secondary antigenic exposure.

Similarly, the protection afforded by antibodies detected in blood donors against Ebola virus infection remains completely unknown. How individuals without any history of haemorrhagic fever acquire specific antibodies to EBOV and what are the biological properties of such antibodies (in particular what is their seroneutralising capacity) deserve further investigations in African populations.

## Supporting Information

S1 Table Associated anti-EBOV IgG seropositivity and potential risk factors stratified by gender (male). (DOCX)

S1 Checklist Checklist of items included in report. (DOC)
