# Supplementary material for: Risk Factors Associated with Ebola and Marburg Viruses Seroprevalence in Blood Donors in the Republic of Congo
Source: PLoS Negl Trop Dis. 2015 Jun 5;9(6):e0003833. doi: 10.1371/journal.pntd.0003833 (PMC4457487; doi:10.1371/journal.pntd.0003833)
Supplement: S1 Table — (DOCX) [file pntd.0003833.s001.docx]

**S1 Table.** Associated anti-EBOV IgG Seropositivity and potential Risk Factors stratified by gender (male)

|  | **Gender (n ;%)** | | **Univariate analysis** | |
| --- | --- | --- | --- | --- |
| **Variables** | **IgG +** | **IgG -** | **OR (95% CI)** | **p-value** |
| **Residence** |  |  |  | 0.19 |
| Urban | 13 (2.4) | 538 (97.6) |  |  |
| Rural | 3 (5.3) | 54 (94.7) |  |  |
| **Residence** |  |  |  | 0.21 |
| Brazzaville | 7 (1.8) | 377 (98.2) |  |  |
| Pointe-Noire | 6 (3.6) | 161 (96.4) |  |  |
| Rural areas | 3 (5.3) | 54 (94.7) |  |  |
| **Age groups, y** |  |  |  | 0.94 |
| 18-29 | 8 (2.7) | 287 (97.3) |  |  |
| 30-39 | 4 (2.2) | 178 (97.8) |  |  |
| 40-49 | 3 (3.4) | 84 (96.6) |  |  |
| 50+ | 1 (2.3) | 43 (97.7) |  |  |
| **Household size** |  |  |  | 0.43 |
| 1-5 | 10 (3.0) | 322 (97.0) |  |  |
| 6-10 | 6 (2.7) | 215 (97.3) |  |  |
| 11+ | 0 (0.0) | 55 (100.0) |  |  |
| **Occupation** |  |  |  |  |
| Health professional | 0 (0.0) | 47 (100.0) |  | 0.24 |
| Office worker | 1 (2.2) | 45 (97.8) |  | 0.84 |
| Military | 3 (3.0) | 96 (97.0) |  | 0.78 |
| Unemployed | 3 (1.9) | 155 (98.1) |  | 0.50 |
| Student | 5 (4.0) | 120 (96.0) |  | 0.28 |
| Hunter | 2 (13.3) | 13 (86.7) | **5.6 (1.4-22.6)** | **0.009** |
| Cultivator | 1 (16.7) | 5 (83.3) |  | 0.14 |
| Farmer | 0 (0.0) | 1 3 (100.0) |  | 0.54 |
| Manual labourer | 1 (1.0) | 98 (99.0) |  | 0.27 |
| **Type of house** |  |  |  |  |
| Modern | 14 (2.6) | 519 (97.4) |  | 0.98 |
| Traditional | 2 (2.7) | 73 (97.3) |  |  |
| **Mosquito net (usage)** |  |  |  | 0.30 |
| Simple | 2 (0.8) | 258 (99.2) | **0.19 (0.1-0.8)** | **0.01** |
| Impregnated | 7 (4.5) | 147 (95.5) |  | 0.08 |
| **Travels** |  |  |  |  |
| Central Africa | 7 (3.6) | 185 (96.4) |  | 0.28 |
| West Africa | 0 (0.0) | 24 (100.0) |  | 0.41 |
| Other countries | 0 (0.0) | 26 (100.0) |  | 0.39 |
| **Exposure to Rodents** |  |  |  | 0.30 |
| Mice | 15 (2.9) | 496 (97.1) |  | 0.28 |
| Rats | 8 (3.8) | 203 (96.2) |  | 0.19 |
| **Exposure to Forest animals** |  |  |  |  |
| Bats | 6 (16.2) | 31 (83.8) | **9.2 (3.5-24.1)** | **<0.001** |
| Monkeys | 2 (12.5) | 14 (87.5) | **5.2 (1.3-21.3)** | **0.01** |
| Birds | 2 (5.0) | 38 (95.0) |  | 0.33 |
| Antelopes | 2 (8.3) | 22 (91.7) |  | 0.07 |
| Snakes | 1 (4.2) | 23 (95.8) |  | 0.63 |
| **Consumption of Forest Animals** |  |  |  |  |
| Bats | 0 (0.0) | 20 (100.0) |  | 0.45 |
| Monkeys | 11 (3.7) | 289 (96.3) |  | 0.11 |
| Birds | 9 (6.3) | 134 (93.7) | **4.1 (1.5-11.02)** | **0.002** |
| Antelopes | 10 (2.9) | 333 (97.1) |  | 0.61 |
| Snakes | 3 (2.4) | 123 (97.6) |  | 0.84 |
